# Early career interview: Balkees Abderrahman

**DOI:** 10.2144/fsoa-2019-0145

**Published:** 2020-01-31

**Authors:** Balkees Abderrahman

**Affiliations:** 1 1155 Pressler, Unit 1354 Houston, TX 77030, USA

**Keywords:** breast cancer, metastases, prediction, statistics

## Abstract

Balkees Abderrahman is the Dallas/Ft Worth Living Legend Fellow of Cancer Research at MD Anderson Cancer Center (TX, USA) and split-site PhD candidate under Model ‘Individuals of Very High Quality’ at the University of Leeds (UK), where she studies cancer. She was one of three finalists of the 2019 Future Science Future Star Award. Here, she tells us about her career to-date.

## Please tell us about yourself

In pursuit of bridging the gap between laboratory and clinic, I was fortunate to join Professor V Craig Jordan's laboratory 4 years ago as an MD with no research experience. Today, my completed clinical research project is the world’s first developed multivariate statistical model to predict the risk of brain metastases in early-stage breast cancer, and to have been internally-and-externally-validated in large patient cohorts. This model permits the medical community to easily generate an individualized 10-year risk prediction score for the development of brain metastases in early-stage breast cancer. My two completed translational research projects are the first to provide comprehensive pharmacological and molecular mechanistic studies on two groups of therapeutics: a fetal estrogen named estetrol that is currently evaluated in clinical trials for the treatment of breast cancer, prostate cancer and use in hormone replacement therapy and estrogen mimics named Selective Human Estrogen Receptor Partial Agonists (BM1-135 and TTC-352) that are currently evaluated in clinical trials for the treatment of endocrine-resistant breast cancer. The on-going translational ‘team effort’ project that I am coordinating challenges the long-standing role of hormones as the fuel to cancer growth. We are investigating the exact opposite; how the female hormone (estrogen) safely kills long-term estrogen-deprived breast cancer, and the male hormone (dihydrotestosterone) safely kills androgen-deprived prostate cancer, to benefit a group of breast and prostate cancer patients. Part of this research investigates how these hormones cause cell death in certain bone, immune and placental cells as part of the developmental biology. We are hoping our research will further the understanding of the molecular mechanisms of this fundamental yet poorly understood cellular stress/death biology, and propose novel switch mechanisms to cell death; so that precision therapeutic advances can be developed and applied to cancers, cardiovascular and neurodegenerative disorders, where this stress biology is involved.

To date, my bibliography exceeds 50 publications, including those in *Nature* and *Science*. I have received the Dallas/Ft Worth Living Legend Fellow of Cancer Research at MD Anderson, and a split-site PhD under model ‘Individuals of Very High Quality’ at the University of Leeds, UK. I have received the Lynn Sage Symposium Scholars Program Award, The European Society for Medical Oncology Grant Award, Women in Tech (Biotech) Scholarship, among others. I am a published author of both literature and medical textbooks, as well as, the US Correspondent for the UK Royal Pharmaceutical Society, and a columnist at Oncology Central.

I have worked with national and international organizations such as: TED, AIESEC, British–Arab Exchange (BAX), The German–Jordanian Friendship Society, Operation Smile, King Hussein Cancer Foundation and Center (KHCRF & KHCRC), and International Federation of Medical Students’ Associations (IFMSA). I was, subsequently, the finalist for the Outstanding Youth Award at the UN from over 86 countries for “*Her outstanding work in tackling some of our greatest social, economic, and environmental challenges*.” I have contributed to several US and UK newspapers on social justice causes like freedom of the press, gender equality, etc. I have also mentored young women and minorities in science.

## What made you choose a career in your field?

Why medicine and science? As a child, I looked at the world with a sense of beauty and wonder and asked ‘too many’ questions. Falling in love with science and medicine was inevitable. Today, that curious child remains within me; when an experiment works, for a moment I get a sense of the forces of nature subdued to my will. This is the romantic – and often untold – side of science. To me, science and medicine are the dynamic ‘yin and yang’. Beyond the edges of petri dishes reaching the walls of clinics, science has always been the muse of clinical practice.

Why cancer research? Cancer is best described by Winston Churchill: “*It is a riddle, wrapped in a mystery, inside an enigma; but perhaps there is a key*.” Cancer is an ever-evolving disease that can affect anyone whether poor or rich, young or old, female or male and the struggle that tags along is gruesome. Although cancer treatment has progressed from a black box to a blueprint, much more needs to be done, to extend and save additional lives. I want to be part of the community that provides solutions and discoveries.

## What are the main highlights of your career so far?

I have mentored four medical school undergraduates (women and minorities); to learn scientific research, or complete a thesis and graduate, or advance professionally, etc.

I have received a funded fellowship in cancer research while getting accepted into a split-site PhD program; fulfilling my dream to be a physician–scientist. I have received several awards for my research. I am a correspondent, columnist, contributor and peer reviewer on medical and scientific topics. The highlight, nonetheless, is being an integral part of the discovery project decoding how the female hormone kills breast cancer and the male hormone kills prostate cancer, inspired from these hormones causing death in bone, immune and placental cells. This novel research aims to introduce two pillars to this stress/death biology: a signature regulation in our models, and a new switch mechanism to cell death aimed at precision therapeutic manipulation.

## What is the most difficult challenge you have encountered in your work & how did you overcome it?

I will start by saying that pursuing scientific research after medical school was not encouraged within my local circle of people. Almost everyone dissuaded me from being where I am today, so I have been busy; fighting the good fight!

Young women, often, do not have a seat on the table because of age bias. Their relative lack of experience is often confused for lacking the capacity to innovate. As an MD with no research background, I was not taken seriously in science despite proving myself. Professional women are constantly put in a cage with bars of gender stereotypes. If you are vocal, you are dangerous. If you do not dress and sound serious, you are not perceived as an intellectual woman. I do all this great work, and at the same time, I happen to love fashion and dressing feminine, but many – within the scientific and medical community – believe that a physician or a scientist should dress plain and look serious.

The capacity to innovate or lead knows no age, or gender, or ‘how you should look like’ boundaries! Science can also be stressful and – at times – tedious. The saving grace to this challenging reality was having an altruistic mentor, Prof. Jordan, who allowed my potential to flourish and my merit to be recognized, and who inspired me to mentor others. This is one shining example of how men and women can be powerful allies.

## What is your favorite publication so far?

I can pick purely scientific publications to impress you, but I will make a different choice here, and I will explain why.

My mentorship article in Science Magazine was so well-received that *Science* decided to launch a new initiative called ‘NextGen Voice: Quality Mentors’ based on it, and featuring it [1].

This piece had a ripple effect; highlighting a new type of mentorship that we need to aspire to and practice, based on the philosophy of personalism. It also led to the recruitment of more women into our lab, who wanted to be mentored by me after they read it, and whose careers were advanced because of that article. These ramifications are a beautiful translation to the power of published words. American author Robert Greene cannot be more correct when he said: “*But the human tongue is a beast that few can master. It strains constantly to break out of its cage*.”

## What are your main aims for the future?

I want to successfully deliver on the aims of the current major translational research project with our national and international collaborators.

I want to stay committed to mentoring more young women and minorities and help them climb up the ladder, despite my often back-breaking workload. I want to keep on giving back, because without my mentor's support, I would not have flourished, and mentorship remains the potent catalyst to evolve from a rough rock to a diamond.

## Where do you hope to see yourself in 5 years?

In a workplace, where I am a team player and a giver. In science and medicine, where I am an innovator and a leader. In life, where I am a student and a teacher.
